# Polypept(o)ide‐Based Core–Shell Bottlebrush Polymers: A Versatile Platform for Drug Encapsulation

**DOI:** 10.1002/mabi.202500083

**Published:** 2025-04-22

**Authors:** Bonan Zhao, Jingyuan Wei, Rüdiger Berger, Lin Jian, Kaloian Koynov, Heyang Zhang, Matthias Barz

**Affiliations:** ^1^ Leiden Academic Center for Drug Research (LACDR) Leiden University Einsteinweg 55 Leiden 2333CC The Netherlands; ^2^ Physics at Interfaces Max Planck Institute for Polymer Research Ackermannweg 10 55128 Mainz Germany; ^3^ Department of Dermatology University Medical Center of the Johannes Gutenberg University Mainz Langenbeckstraße 1 55131 Mainz Germany

**Keywords:** “grafting‐from” synthesis, core–shell bottlebrush polymers, drug delivery, polypept(o)ide

## Abstract

Cylindrical bottlebrush polymers (CBPs) enable the precise adjustment of nanoparticle properties such as size, shape, and functionality exclusively by polymer synthesis. In addition, block copolymer side chains enable direct access to core–shell structure. In this study, the synthesis of polypept(o)ides‐based core–shell CBPs is presented through a “grafting‐from” strategy. While, poly‐lysine (pLys) serves as the backbone, poly(γ‐benzyl‐l‐glutamic acid)‐block‐polysarcosine (pGlu(OBn)‐*b*‐pSar) copolymers form the side chains. This approach enables the synthesis of core–shell nanoparticles, referred as core–shell brushes (CSBs), with hydrodynamic radius (*R*_h_) from 17 to 70 nm, and molecular weights (1320–4000 kg mol^−1^) with dispersity indices ≈1.3 as determined by size‐exclusion chromatography. Dasatinib is chosen as a drug molecule model to explore the potential of such synthetical CSBs as a platform for drug encapsulation by *π*–*π*‐interactions. An overall loading efficiency of 10% is achieved, which also displayed sustained release within 72 h, cellular uptake into human glioblastoma (U‐87 MG) cells, and drug‐related therapeutic efficacy. While drug release can be further optimized by covalent drug attachment, these results establish a strong foundation for the use of CSBs in nanomedicine.

## Graphical abstract

This study developed polypept(o)ide‐based core–shell brushes (CSBs) with tunable sizes (*R*_h_: 17–70 nm) for hydrophobic drug delivery. CSBs achieved 10% dasatinib loading through *π*–*π* interactions, enabling sustained release over 72 h. Notably, CSB‐dasatinib complexes showed comparable cell viability reduction to the free drug in U‐87 MG cells, demonstrating its potential for advanced cancer therapy.

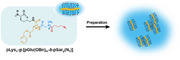

## 1 Introduction

Over the past decades, several nanosized drug delivery systems have advanced chemotherapeutics from preclinical toward clinical studies.^[^ ^1^ ^]^ Nanoparticle‐based drug delivery systems are well known to enhance bioavailability and can improve pharmacokinetics of conjugated therapeutic agents.^[^ ^2^ ^]^ These systems offer extended plasma half‐life due to size‐dependent slower clearance in vivo.^[^ ^3^, ^4^ ^]^ Moreover, they facilitate passive tumor accumulation in well‐vascularized tumors, e.g. Kaposi sarcoma,^[^ ^5^ ^]^ leveraging the enhanced permeability and retention (EPR) effect.^[^ ^6^ ^]^

Linear and mikto‐arm star synthetic block copolymers have been extensively utilized to fabricate nanosized delivery systems.^[^ ^7^, ^8^, ^9^, ^10^, ^11^ ^]^ While low‐dimensional polymers such as linear block copolymers can self‐assemble into an array of different aggregates in a block selective solvent, they often display dynamic behavior and high sensitivity to external conditions and require core‐crosslinking for stabilization. Instead, high‐dimensional polymers such as cylindrical bottlebrush polymers (CBPs) with amphiphilic diblock copolymer side chains form stable core–shell structures.^[^ ^12^, ^13^ ^]^ CBPs are characterized by polymeric side chains densely grafted onto a linear polymer backbone.^[^ ^14^, ^15^, ^16^ ^]^ This results in a highly stretched main chain due to steric repulsion,^[^ ^17^, ^18^, ^19^, ^20^ ^]^ which can be fine‐tuned through variations in microstructure (block or gradient copolymers) into virus‐like^[^ ^21^ ^]^ or patchy morphologies.^[^ ^22^ ^]^

Therefore, these functional CBPs exhibit diverse architectures, including core–shell,^[^ ^23^ ^]^ bottlebrush block copolymers,^[^ ^24^, ^25^, ^26^ ^]^ Janus,^[^ ^27^, ^28^ ^]^ or brush‐on‐brush structures.^[^ ^29^ ^]^ Such versatile architectures make CBPs a promising platform for a range of applications in material science and biomedicine, for drug delivery,^[^ ^20^, ^30^, ^31^ ^]^ diagnostics,^[^ ^32^ ^]^ and beyond. For instance, Wang and co‐workers designed multifunctional unimolecular micelles formed by amphiphilic bottlebrush‐like grafted block copolymers with theragnostic functions of bright far red/near infrared fluorescence imaging and anticancer (doxorubicin) drug delivery.^[^ ^32^ ^]^ Jiang and co‐workers synthesized polyaspartamide‐based disulfide‐containing brushed polyethylenimine derivatives, via click chemistry and showed its potential in pDNA delivery as polyplexes.^[^ ^33^ ^]^ Similarly, the Johnson lab reported triblock bottlebrush copolymers composed of polylactic acid, polyethylene glycol (PEG), and poly(*N*‐isopropylacrylamide) (PNIPAM), synthesized through ring‐opening metathesis polymerization (ROMP).^[^ ^34^ ^]^ Müllner and collaborators developed UV‐responsive core–shell nanodiscs from tadpole‐like bottlebrush polymers with poly(ethyl glyoxylate) chains.^[^ ^35^ ^]^

Together with the group of Manfred Schmidt, we previously reported worm‐like core–shell bottlebrush polymers with poly[*N*‐(6‐aminohexyl)methacrylamide] (PAHMA) backbones, pLys cationic cores, and pSar as the side chain shells, for siRNA delivery.^[^ ^20^ ^]^ Transitioning from PAHMA to polypeptide‐based systems allows for the synthesis of CBPs completely based on endogenous amino acids.^[^ ^19^ ^]^ The pSar side chains offer reduced immunogenicity compared to PEG,^[^ ^36^, ^37^, ^38^, ^39^ ^]^ while pLys can act as a scaffold for the grafting‐from synthesis of CBPs by *N*‐Carboxyanhydride (NCA) ring‐opening polymerization (ROP), combining the benefits of polypeptides and polypeptoids.^[^ ^40^, ^41^ ^]^ Besides, the synthesis of polypeptoids by ROP proceeds in the absence of the activated monomer mechanism,^[^ ^42^ ^]^ which enables precise control over chain length and high grafting densities, making it particularly interesting for the synthesis of CBPs with high aspect ratio and high grafting density.^[^ ^43^, ^44^ ^]^

In this study, we synthesized core–shell CBPs by polymerizing γ‐benzyl‐l‐glutamic acid‐NCA (Glu(OBn)‐NCA) and Sar‐NCA from a pLys backbone. The core–shell CBPs, namely pLys_250_ *‐g*‐[pGlu(OBn)_25_‐*b*‐pSar_204_(N_3_)] (**CSB‐L**) and pLys_250_ *‐g*‐[pGlu(OBn)_5_‐*b*‐pSar_50_(N_3_)] (**CSB‐S**), are characterized using size exclusion chromatograph (SEC), proton nuclear magnetic resonance (^1^H NMR), diffusion‐ordered spectroscopy (DOSY‐NMR), multi‐angle dynamic laser light scattering (MADLS), static laser light scattering (SLS) and atomic force microscopy (AFM). Driven by the intermolecular π‐πinteractions between benzyl groups in the hydrophobic pGlu(OBn) block and hydrophobic drugs, a stable encapsulation can be achieved. Here, we chose Dasatinib, a ATP‐competitive protein tyrosine kinase inhibitor used in the therapy of chronic myelogenous leukemia and acute lymphoblastic leukemia.^[^ ^45^ ^]^ We evaluated the drug loading by dual centrifugation, release profile, and drug activity on human glioblastoma cell line (U‐87 MG) to assess the potential of CSBs for drug delivery.

## 2 Results and Discussion

### 2.1 Pept(o)ides‐Based Core–Shell Brushes: Controlled Syntheses and Characterization

**Scheme 1:**
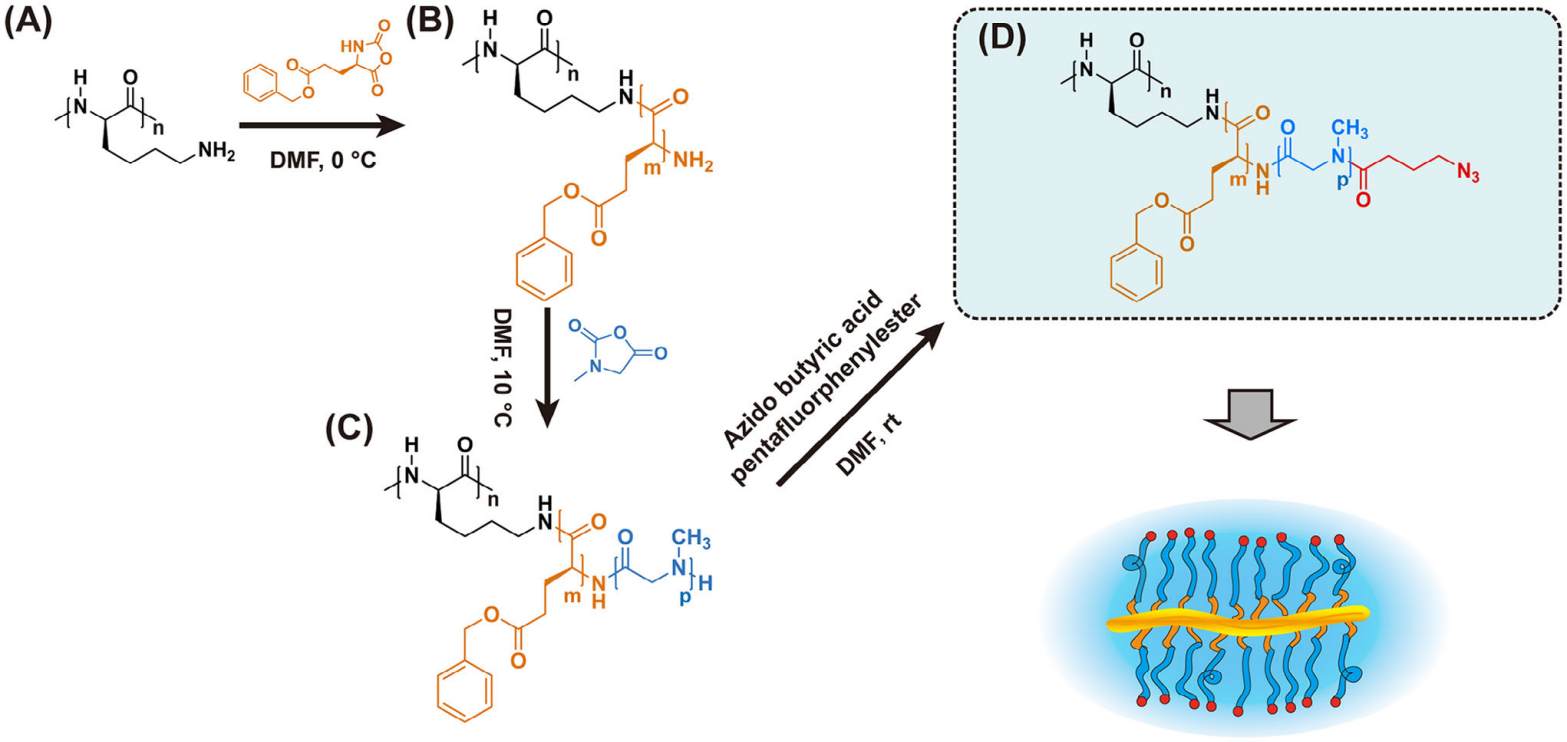
Synthetic route of polypept(o)ide core–shell bottlebrush polymers. A) pLys_n_, B) pLys_n_ *‐g*‐pGlu(OBn)_m_, C) pLys_n_ *‐g*‐[pGlu(OBn)_m_‐*b*‐pSar_p_], D) pLys_n_ *‐g*‐[pGlu(OBn)_m_‐*b*‐pSar_p_(N_3_)].

The synthesis of CSBs with pGlu(OBn)‐*b*‐pSar block copolymers as side chains were performed by sequential ROP of the corresponding NCAs (see **Scheme** **1**). All NCAs were synthesized by the Fuchs–Fathing method using diphosgene and were intensively purified. While Glu(OBn) NCAs were purified by recrystallization, the sarcosine NCA could be purified by sublimation under a high vacuum.^[^ ^43^, ^46^, ^47^ ^]^ Yields and melting points were in line with published literature (see Experimental Section).^[^ ^48^ ^]^ The silver nitrate test was negative for all purified NCAs. All synthesized NCAs have been stored at −80 °C over months without any detectable degradation or oligomerization.

**Figure 1:**
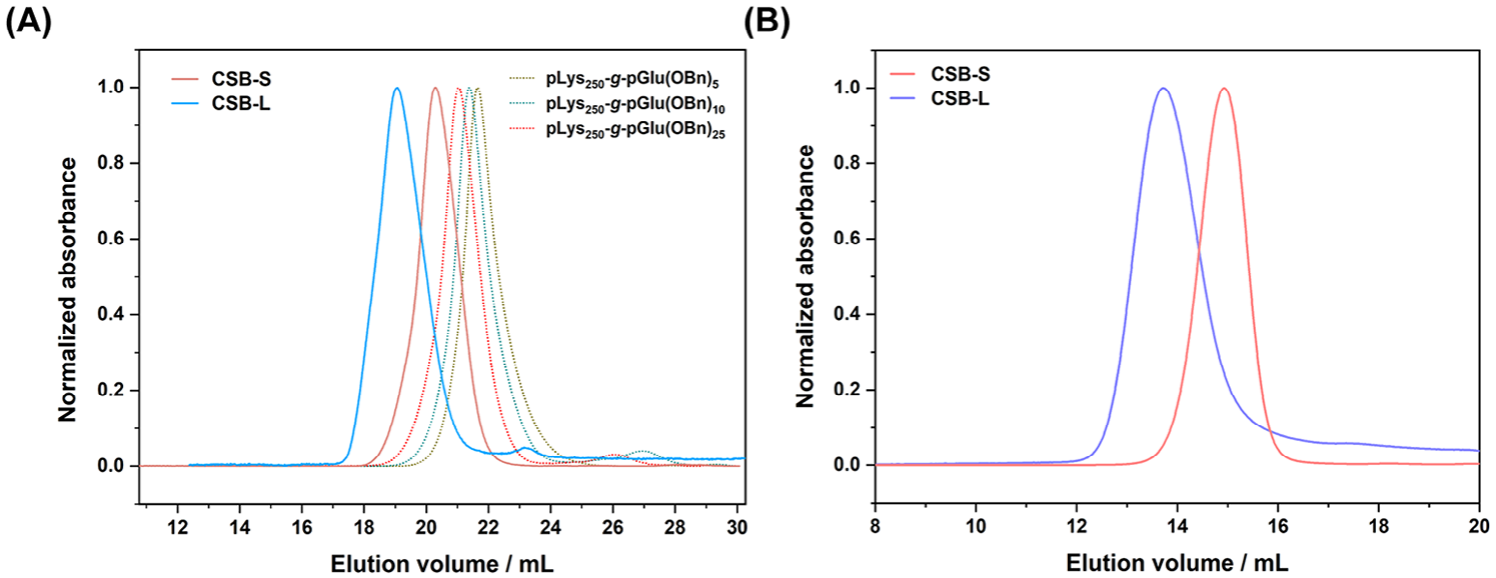
Size‐exclusion chromatography (SEC) traces of pLys_250_‐*g*‐pGlu(OBn)_5_ (dashed olive line), pLys_250_‐*g*‐pGlu(OBn)_10_ (dashed blue line), pLys_250_‐*g*‐pGlu(OBn)_25_ (dashed pink line) in HFIP A). SEC traces of pLys_250_‐*g*‐[pGlu(OBn)_25_‐*b*‐pSar_204_(N_3_)] (CSB‐L) and pLys_250_‐*g*‐[pGlu(OBn)_5_‐*b*‐pSar_50_(N_3_)] (CSB‐S) in HFIP (A) and in PBS B).

We employed a pLys with a chain length of ≈250 as a model backbone comparable to our previous study.^[^ ^19^ ^]^ All polymerizations were conducted at an NCA concentration ([M]_0_ = 100 mg mL^−1^) in purified DMF (< 60 ppm water) and full monomer conversion could be observed by FT‐IR by the disappearance of the characteristic carbonyl stretching band of the anhydride group of the NCA ring at 1788 and 1858 cm^−1^. pGlu(OBn), as “hydrophobic core”, was grafted from the pLys at first with different chain lengths (defined degree of polymerization (*DP*_n_) ≈5–25). In the case of pGlu(OBn), the polymerization had to be performed at 0 °C to avoid backbiting of the reactive chain end (intramolecular amide bond formation) as reported by Heise and co‐workers.^[^ ^49^ ^]^ However, with increased [M]_0_/[I]_0_ ratios from 5 to 25, a minor second elution peak following the main monomodal and narrow brush peak can be observed in hexafluoroisopropanol (HFIP)‐SEC (**Figure** **1**A), which may arise from water or dimethylamine initiation in DMF. This homopolymer impurity, however, can be easily removed by spin filtration, SEC, or reprecipitation. Interestingly, a single monomodal and symmetrical elution peak of brush polymer with shortest target side chains (≈5) was observed and no linear polymer from the activated monomer mechanism was detected (Figure 1A, dashed olive line). Nevertheless, the low molecular weight impurities can be removed before the addition of the Sar‐NCA.

To ensure proper shielding and stealth‐like properties of CBPs, we set the pSar side chain length as a “hydrophilic shell” to be ≈8–10 times longer than pGlu(OBn).^[^ ^50^, ^51^, ^52^ ^]^ The pGlu(OBn) blocks of the pLys_250_‐*g*‐pGlu(OBn)_5_ and pLys_250_‐*g*‐pGlu(OBn)_25_ macroinitiators were applied to pSar shell grafting. The ROP of the Sar NCA was performed in purified DMF at 10 °C, combining controlled polymerization with fast polymerization kinetics. After full monomer conversion has been observed by FT‐IR, the end groups of individual side chains were capped with azido‐butyric acid pentafluorophenyl ester to introduce azide end groups to the surface of CBPs. This strategy allows for further conjugation of fluorescent or radioactive markers or bioactive compounds by strain‐promoted azide alkyne coupling (SPAAC) employing DBCO (Scheme 1).^[^ ^53^ ^]^

SEC analysis performed in HFIP showed symmetric and monomodal molecular weight distributions for both polypept(o)ide‐based CSBs with molecular weight (*M*_w_) over 1000 kg mol^−1^, with low to moderate polydispersity values (*Đ* = 1.2–1.3) (as depicted in Figure 1A; Figure S3, Supporting Information and **Table** **1**). In line with HFIP‐SEC analysis, aqueous SEC analysis in phosphate‐buffered saline (PBS) confirmed the expected shift in elution volumes for CSB‐L and CSB‐S, underlining the controlled polymerization across different block side chains (Figure 1B). Only the CSB‐L displayed a low molecular weight tailing in SEC analysis in both solvents, which can be removed by dialysis, spin‐filtration, or precipitation as reported before.^[^ ^20^ ^]^ The uniformity after purification was ensured by a single CBP species observed in ^1^H‐DOSY NMR (Figures S1 and S2, Supporting Information).

**Table 1:** Analytical results of the core–shell brush polymer.

Polymer	*M_n_* [kg mol^−1^]^a)^	*M_w_* [kg mol^−1^]^b)^	*M_n_* [kg mol^−1^]^c)^	*Ð* (SEC)^d)^	*R*_h_ [nm]^e)^	*Ð* (Size)^e)^	ζ‐potential [mV]^f)^	*R*_h_ [nm]^g)^	*R*_g_ [nm]^b)^	*R_g_/R_h_*
pLys_250_‐*g*‐[pGlu(OBn)_5_‐*b*‐pSar_50_(N_3_)] **CSB‐S**	1200	1320	1100	1.2	16	0.08	−6.3	17	18	1.1
pLys_250_‐*g*‐[pGlu(OBn)_25_‐*b*‐pSar_204_(N_3_)] **CSB‐L**	5000	4000	3080	1.3	60	0.19	−5.2	70	72	1.0

^a)^ Determined by ^1^H NMR;

^b)^ Determined by SLS;

^c)^ Calculated from SLS data;

^d)^ Determined by HFIP‐SEC relative to PMMA standards;

^e)^ Determined by Single‐angle DLS at 173°;

^f)^ Determined in 10 mm HEPES buffer in disposable folded capillary cells at 25 °C;

^g)^ Determined by multi‐angle DLS at 26°, 58°, 90°, and 122°.

The pGlu(OBn) and pSar side chain lengths were determined by end‐group analysis via ^1^H‐NMR spectroscopy, indicating successful synthesis of CSBs with controlled pSar side chain lengths of 204 (DP*_expected_* 200) and 50 (DP*_expected_* 60) and pGlu(OBn) blocks with chain lengths of 25 (DP*_expected_* 25) and 5 (DP*_expected_* 5), respectively as depicted in Table 1, Figures S1 and S2 (Supporting Information). In detail, the integral of the methyl group in the azido moiety (3.2–3.4 ppm (m, 2H)) was related to the pSar and pGlu(OBn) signal (─CH_2_─CH_2_─**CH_2_** ─N_3_) in the ^1^H NMR spectra of individual polymers. We can, however, not ensure that all side chains grew identically. To further analyze the side chain length and dispersity and time‐dependent stability of CSBs, a protease assay was conducted using natural protease B from *Streptomyces griseus* (SGPB), a small protease (185 amino acids, *R*_h_ ≈1.6 nm) with broad hydrolytic activity.^[^ ^54^ ^]^ CSB co‐incubation with SGPB at 37 °C revealed degradation over 21 d, as evidenced by the decay of the CBP peak and the emergence of peaks at higher elution time in HFIP‐SEC (Figure S4, Supporting Information). Interestingly, the polypeptide segments degraded, while pSar side chains remained intact during the enzymatic degradation and can be analyzed by HFIP‐SEC. Relative to pSar standards an average *DP* of ≈200^[^ ^43^ ^]^ and a polydispersity of 1.07 was determined for the obtained degradation products. Complementary ^1^H NMR analysis of CSB‐S in D_2_O and DMSO‐*d*_6_ provided further insight into core hydrophobicity: benzyl peaks (7.0–7.5 ppm) broadened in D_2_O, indicating a hydrophobic benzyl‐core structure (Figure S1, Supporting Information), while disappearing benzyl signals in CSB‐L suggested aggregation of the pGlu(OBn)_25_ segment in aqueous environments (Figure S2, Supporting Information).

**Figure 2:**
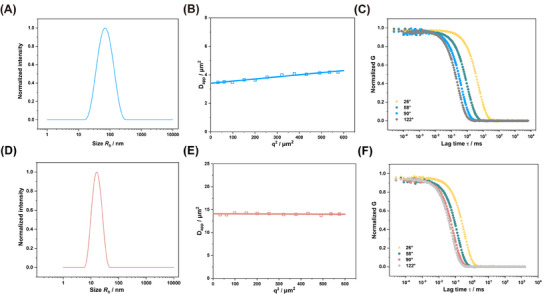
Representative size distribution of CSB‐L A) and CSB‐S D) at 90°. Multi‐angle DLS analysis of CSB‐L B) and CSB‐S E) in PBS from 26° to 122°. Autocorrelation functions CSB‐L C) and CSB‐S F) were given for a representative measurement angle at 26°, 58°, 90°, and 122°.

To characterize the hydrodynamic radius of CSBs in an aqueous solution (PBS), we employed different light scattering methods. First, single‐angle DLS measurements were performed at 173° (Malvern ZetaSizer Nano ZS). As outlined in Table 1, CSB‐S and CSB‐L exhibited distinct hydrodynamic radii (*R*_h_) of 16 and 60 nm, with a polydispersity index (PDI) of 0.08 and 0.19, respectively. For CSB‐S, the hydrodynamic radius was in line with the one obtained through multi‐angle DLS (ALV/CGS‐8F SLS/DLS 5022F Goniometer) (as seen in Table 1, **Figure** **2**D), since for such short CBP, its angle dependency is absent (Figure 2E) while a slight angle dependency of *D*_app_ for CSB‐L (Figure 2B). Autocorrelation function (ACF) analysis indicates the absence of aggregates or high molecular weight impurities in the sample (Figure 2C,F), while the average *R*_h_ was derived as 17 and 70 nm from the CONTIN fit of the autocorrelation curve at 26°, 58°, 90°, and 122° angles (Table 1). Both CSBs displayed neutral ξ‐potentials of −6.3 and −5.2 mV, confirming the successful end‐capping with azido groups on the secondary amine of terminal sarcosine (Table 1; Figures S1 and S2, Supporting Information).

Next, SLS measurements were carried out to determine the weight average molecular weight *M*_w_ (in Table 1) for both CSBs. We determined the specific refractive index increment (dn/dc) to be 0.176 cm^3^ g^−1^ in PBS by differential refractometry. The *M*_w_ was 1320 kg mol^−1^ for CSB‐S and 4000 kg mol^−1^ for CSB‐L (Table 1). Interestingly, the *M*_w_ for CSB‐S aligns well with the molecular weights calculated from the *DP*_n_ of main and side chains, while the *M*_w_ of CSB‐L from SLS was ≈80% of the value derived from ^1^H NMR data, indicating a lower pSar grafting density in CSB‐L of ≈80%. Additionally, we determined the radius of gyration (*R*_g_) to be 18 nm for CSB‐S with shorter pGlu(OBn)‐*b*‐pSar side chain length, and 72 nm for CSB‐L with longer side chains. The *R*_g_/*R*_h_ ratio 1.0 to 1.1 confirmed an ellipsoidal morphology, which is consistent with the structural features of CSB‐S observed via AFM (Figure S6, Supporting Information).

Finally, fluorescence correlation spectroscopy (FCS) was employed to evaluate both CSBs labeled with Alexa Fluor 647 in PBS and non‐diluted human serum (Table 1). Following 1 h incubation in human serum, the *R*_h_ of CSB‐S remained identical (18 vs 18 nm in PBS), due to the shielding of pSar preventing from proteins’ absorption (Figure S5B, Supporting Information). In contrast, CSBs with extended pGlu(OBn) side chains exhibited detectable aggregation in human serum (Figure S5A, Supporting Information). Although a high degree of polymerization (*DP* > 200) of pSar was grafted to enhance steric repulsion and avoid protein corona formation, the extended chain length of pGlu(OBn) and increased hydrophobic “core regions” seem to induce pronounced protein binding. Since the temperature was precisely controlled to avoid side reactions in pGlu(OBn) formation and the same conditions can be applied to linear block copolymer synthesis in solution, the reason for the observed phenomenon cannot be identified and will be investigated in more detail in future research. The CSB‐L, however, could not be used for drug encapsulation or biological studies and we proceeded only with CSB‐S.

### 2.2 Drug Loading of Core–Shell Brush with Dasatinib (DAS): Formulation and Characterization

**Figure 3:**
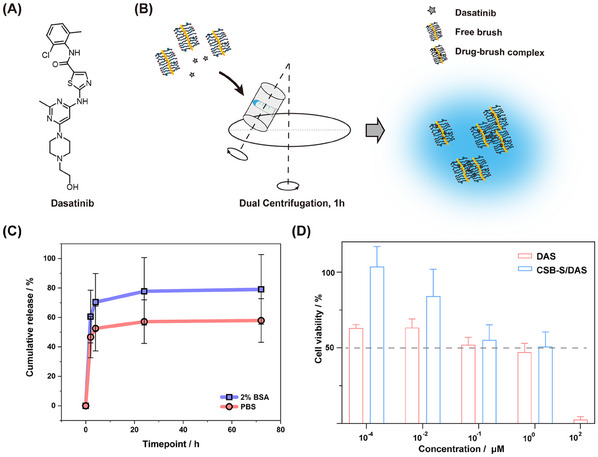
Dasatinib A), was chosen as a drug molecule model and loaded onto CSB‐S via dual centrifugation B). Cumulative release profile C) of DAS from drug/brush complex was studied by exposing drug/brush complex in 2% BSA/PBS or PBS at 37 °C. At defined timepoint (2 h, 4 h, 24 h, 48 h), samples were collected and analyzed using the established analytic assay based on UPLC (n=3). Cell viability D) was assessed using MTT assay after 72‐h exposure to free DAS and CSB‐S/DAS respectively (n = 9). All experiments were implemented in triplicates.

To explore the potential of such core–shell brush being a platform for drug delivery, Dasatinib (DAS), an ATP‐competitive protein tyrosine kinase inhibitor used in the therapy of chronic myelogenous leukemia (CML) and acute lymphoblastic leukemia (ALL) was chosen, and loaded by dual centrifugation (DC), following the recently reported protocol by N.J.K. Dal^[^ ^51^ ^]^ et al. (**Figure** **3**A,B). The loading efficiency was 8.8 ± 3.2%, according to the established quantification assay based on UV–vis spectroscopy (Figure S7, Supporting Information). The low loading efficiency is likely due to the short pGlu(OBn) block length of 5 units on average within CSB‐S. Previous studies have demonstrated that when brushes form inter‐brush loops, the drug loading efficiency is significantly enhanced due to the structural features that facilitate drug encapsulation.^[^ ^55^ ^]^ In our case, however, the drug loading efficiency remains below 10%.

Upon drug loading and quantification, drug release kinetics for the DAS‐loaded CSB‐S were determined in PBS with or without the addition of 2% (w/v) bovine serum albumin (BSA) at 37 °C according to a previous study.^[^ ^53^ ^]^ An initial burst release, ≈40%, was detected within the first 2 h, followed by a sustained release over the subsequent 70 h, reaching a total of 58% in PBS, calculated based on UPLC calibration curve (Figure S8, Supporting Information). The burst release indicates that not all drugs penetrated the pGlu(OBn) core and may remain at the interphase of pSar and pGlu(OBn) or even in the pSar corona as recently demonstrated by Luxenhofer and coworkers for polyoxazoline micelles.^[^ ^56^ ^]^ The presence of BSA (2%) led to a modest acceleration in drug release, likely due to the transfer of DAS to BSA and the formation of drug‐BSA complexes through hydrophobic interactions,^[^ ^57^ ^]^ resulting in ≈80% cumulative release at 72 h, though this increase was not statistically significant compared to the counterpart in PBS (Figure 3C).

### 2.3 Biological Evaluation of Core–Shell Brushes with and without Drug Loading

Further, we visualized the interactions between CSB‐S/DAS and cells, by exposing drug‐loaded CSB‐S to the human glioblastoma cells (U‐87 MG cells) for 4 h (Figure S10, Supporting Information). Due to the notable shielding effect of pSar, the cellular internalization of AF647‐labeled CSB‐S was rather low but still much more notable than the cells exposed to CSB‐S/DAS. Next, we evaluated the effect of DAS on cell viability and compared free to CSB‐S bound drugs. After 72 h exposure, the IC_50_ value of free DAS was found 0.1 µm, which was comparable to the value (1.5 µm) in the previous study with 72 h‐incubation on U‐87 MG cell line.^[^ ^58^ ^]^ The CSB‐S/DAS exhibited an IC_50_ ≈0.6 µm, which was comparable to that of the free drug (0.1 µm) (Figure 3D). Since DAS exerts its inhibitory effects by targeting the Src family of kinases inside cells,^[^ ^58^, ^59^ ^]^ the delay in cellular toxicity seems reasonable. Nevertheless, the reduction in cancer cell viability by CSB‐S/DAS reaches levels comparable to the free drug DAS. Besides, the CSB‐S did not show any influence on cell viability in the concentration range of 0.1–10 nm (Figure S11, Supporting Information).

## 3 Conclusion

In conclusion, we have successfully demonstrated the controlled synthesis and characterization of polypept(o)ide‐based CSBs via a “grafting‐from” strategy and one‐pot synthesis. These CSBs are composed of polysarcosine and benzyl‐protected polyglutamic acid as side chains, with polylysine serving as the backbone. We obtained well‐defined core–shell brushes with varying *R*_h_ from 17 to 70 nm, molecular weight between 1,320 and 4,000 kg mol^−1^, and dispersity indices of ≈1.3. Cellular toxicity assessment using an MTT assay in U‐87 MG cells revealed that pLys_250_‐*g*‐[pGlu(OBn)_5_‐*b*‐pSar_50_(N_3_)] did not reach the IC_50_ value, suggesting that these CSBs can function can be employed for the design of drug formulations. Furthermore, CSB‐S has been applied to the encapsulation of DAS. The drug‐loaded CSB‐S/DAS displayed controlled drug release profiles in both PBS and BSA solutions, alongside sustained delivery characteristics, achieving a comparable reduction of cancer cell viability like the free drug in vitro. While our in vitro studies demonstrate an initial drug release, this characteristic may have important in vivo implications. Drug release could be advantageous in conditions requiring immediate therapeutic action in blood, such as acute leukemia or bacterial infections or local therapies after surgery. The subsequent sustained release phase may help maintain therapeutic drug concentrations over an extended period. Further in vivo studies will be necessary to evaluate the pharmacokinetics, biodistribution, and therapeutic efficacy of this system in different disease models. Nevertheless, the present study lays the foundation for core–shell CBPs based on polypept(o)ides.

## 4 Experimental Section

### Materials

All reagents and solvents were purchased from Sigma‐Aldrich and were used as received unless specified otherwise. *N, N*‐Dimethylformamide (DMF) was acquired from Fisher Scientific (99.8% purity, further dried over molecular sieves) and subjected to repetitive freeze‐pump‐thaw cycles for purification (water content, < 50 ppm). Milli‐Q (MQ) water was generated using a MILLI‐Q Reference A+ system, ensuring a resistivity of 18.2 MΩ·cm^−1^ and total organic carbon of < 5 ppm. Hexafluoroisopropanol (HFIP) and potassium trifluoroacetate were sourced from Fluorochem. Deuterated solvents were obtained from Deutero GmbH and utilized without further treatment. Poly‐l‐lysine trifluoroacetate was ordered from Alamanda Polymers, Inc. Dasatinib (DAS, 99.99%, HPLC level) was purchased from SelleckChem.

Dulbecco's Modified Eagle's Medium/F12 (DMEM/F12), trypsin/EDTA, trypan blue, and MTT (3‐(4,5‐Dimethylthiazol‐2‐yl)‐(4‐2,5‐diphenyl tetrazolium bromide) were bought from Thermo Fisher Scientific (Landsmeer, The Netherlands). Dulbecco's Phosphate‐Buffered Saline (no calcium, no magnesium, (DPBS[‐]), l‐glutamine, and PEN‐STREP (10 000 U mL^−1^ penicillin, 10 000 U mL^−1^ streptomycin) were purchased from Lonza Bioscience (Verviers, Belgium). Fetal bovine serum was obtained from SERANA (Brandenburg, Germany). Human serum was provided by the transfusion center of the Medical Department of the Johannes Gutenberg University Mainz. The serum was pooled of six healthy donors.

### Characterization Methods

Proton nuclear magnetic resonance (^1^H NMR) spectra were acquired on a Bruker Avance II 400 at room temperature operating at 400 MHz. DOSY NMR spectra were recorded on a Bruker Avance II 400 using a bipolar pulse program (stebpgp1s) with d20 = 0.2 and p30 = 2750 µs for gradient amplitudes from 5% to 95%. Spectral calibration was performed using solvent signals, and the analysis was conducted using MestReNova version 14.0.0 from Mestrelab Research S.L.

Melting point: The melting point of Sarcosine‐NCA and Glu(OBn)NCA were determined using a METTLER FP62 (METTLER WAAGEN GMBH) melting point apparatus. Approximately 2‐3 mg of the recrystallized compound was placed in a sealed capillary tube. The measurement was conducted under atmospheric pressure with a controlled heating rate of 1 °C/min, starting from room temperature. The melting point was recorded as the onset temperature at which the sample began to liquefy. All measurements were performed in duplicate to ensure reproducibility.

Attenuated total reflectance Fourier transform infrared (ATR‐FTIR) spectroscopy was carried out on an FT/IR‐4100 instrument (JASCO Corporation) equipped with an ATR sampling accessory (MIRacle, Pike Technologies). IR spectra were analyzed using Spectra Manager 2.0 (JASCO Corporation). NCA polymerization progress was monitored by FT‐IR spectroscopy, with the completion of polymerization confirmed by the disappearance of carbonyl peaks at 1858 and 1788 cm^−1^.

Size‐exclusion chromatography (SEC) was conducted using a Jasco SEC setup operating at a flow rate of 1.0 mL·min^−1^ and a temperature of 40 °C. Two eluents were used, including HFIP and DPBS. HFIP SEC: HFIP served as the eluent, containing potassium trifluoroacetate (3 g·L^−1^). The column material was sourced from PSS Polymer Standards Service GmbH, featuring modified silica gel as the column material (PFG columns, particle size: 7 µm, porosities: 100 and 1000 Å). For polymer detection, a UV detector (Jasco UV‐2075+) at a wavelength of λ = 230 nm was utilized with toluene employed as an internal standard. The elution data were evaluated using PSS WinGPC (PSS Polymer Standards Service GmbH). Aqueous SEC (flow rate at 0.5 mL·min^−1^): DPBS from Sigma–Aldrich served as the eluent and TSKgel GMPWXL 808025 as a mixed bed scouting column was used for aqueous water‐soluble linear polymers from Tosoh Corporation. An UV detector from Jasco UV‐2075+ at λ = 230 nm was also utilized. CSB‐L was purified via preparative SEC using a Sepharose 4 FF XK 16/70 column (flow 0.5 mL min^−1^). The fraction from 98 to 126 mL was collected, concentrated using Amicon Ultra centrifugal filter devices (100 kDa, 4000 g), and filtered through 0.22 µm filters.

Enzymatic Degradation. Protease (from *Streptomyces griseus*, TypXIV, Sigma Aldrich) was dissolved in 0.5 mL buffer (c = 12 mg mL^−1^). The buffer (pH = 7) consists of 10 mM sodium acetate and 5 mM calcium acetate. CSB‐L was dissolved in 0.5 mL buffer to a final concentration of 8 mg mL^−1^. These two solutions were then mixed to yield a final CSB‐L concentration of 4 mg mL^−1^ and incubated in a Eppendorf ThermoMixer C at 37 °C and 500 rpm. At defined time points, an aliquot was sampled, lyophilized and analyzed by HFIP‐SEC. The chain length was determined by HFIP‐SEC relative to polysarcosine standards.^[^ ^43^ ^]^

Single‐angle dynamic light scattering (DLS) measurements and ζ‐potential measurements were conducted using a ZetaSizer Nano ZS instrument (Malvern Instruments Ltd, Worcestershire, UK) equipped with a He‐Ne laser (λ = 632.8 nm) as the incident beam. These measurements were performed at 25 °C and a detection angle of 173°. All solutions were prepared at 0.3 mg mL^−1^ in PBS and filtered into the cuvettes through Millex‐GV filters with a pore size of 0.2 µm. The ζ‐potential was determined in 10 mm HEPES buffer (pH 7.4).

Multi‐angle dynamic light scattering (DLS) and static light scattering (SLS). All solutions were filtered into the cuvettes through Millex‐GV filters with a pore size of 0.2 µm. DLS measurements were conducted at 20 °C using a Uniphase He/Ne Laser (22.5 mW output power at λ = 632.8 nm) and an ALV/CGS‐8F SLS/DLS 5022F goniometer with eight simultaneously operating ALV 7004 correlators and eight ALV/High QEAPD avalanche photodiode detectors. Autocorrelation curves were analyzed using ALV‐Correlator Software, and the reported hydrodynamic radius represents the inverse z‐averaged hydrodynamic radius, <1/R_h_>_z_^−1^. SLS analysis was carried out at 8 different scattering angles (26°–122°) for varying concentrations (50–400 µg·mL^−1^). A Zimm plot was constructed using ALVStat 4.31 software (ALV, Germany), yielding information about the weight average molecular weight (*M*_w_), the second virial coefficient (A_2_), and the square root of the z‐averaged mean square radius of gyration (*R*_g_ = <S^2^>_z_^1/2^), employing a dn/dc value of ≈0.176 mL g^−1^ for pSar brushes in PBS or MeOH.^[^ ^20^ ^]^

Atomic force microscopy (AFM) measurements were conducted in the air using a Dimension ICON (Bruker) in tapping mode (OTESPA (Bruker), back‐side coated with a nominal resonance frequency of 300 kHz and a spring constant of 26 N m^−1^). We prepared samples by drop‐casting a particle solution (20 µL; 1 mg·L^−1^ in MQ water) onto freshly cleaved mica substrates. The prepared samples were allowed to dry overnight at room temperature in a vacuum of 1 kPa. AFM images were analyzed using Gwyddion software.

Fluorescence correlation spectroscopy (FCS). FCS experiments were performed using a commercial confocal LSM 880 microscope (Carl Zeiss, Jena, Germany) equipped with a C Apochromat 40×/1.2 W (Carl, Zeiss, Jena, Germany) water immersion objective. A HeNe laser (λ = 633 nm) fiber coupled to the LSM 880 was used to excite the Alexa Fluro 647 dye. The emission light in the spectral range 655–699 nm was detected using a spectral detection unit (Quasar, Carl Zeiss). 200 µL of solution was added per well to the 8‐well polystyrene‐chambered cover glass (Nunc Lab‐Tek, Thermo Fisher Scientific, Waltham, MA). The confocal detection volume was placed at 30 µm above the glass coverslip and a series of 20 measurements, 10 s each, were performed at room temperature (23 °C). The obtained experimental autocorrelation curves were fitted with the following analytical model function: whereby *f*_*T*_ and *τ*_*T*_ are the fraction and the decay time of the triplet state, *N* is the average number of diffusing fluorescence species in the observation volume, *τ*_*Di*_ is the diffusion time of the *i*‐th type of species, *f*_*i*_ is the fraction of component i (1 ≤ *i* ≤ m), and *S* is the structure parameter, *S* = *z*_*0*_/*r*_*0*_, where *z*_*0*_ and *r*_*0*_ represent the axial and radial dimensions of the observation volume. The data was fitted with the ZEN 3.0 software (Carl Zeiss, Jena, Germany) using a 2 diffusion component model, *τ*_*D1*_ was fixed, and calibrated by 10 nM Alexa Fluro 647 in PBS. The fitting yielded the fraction of free dye (*f*_*1*_) and CSB (*f*_*2*_), *τ*_*D2*_, *N*, and *S*. The diffusion coefficients of the species *D*_*i*_ are related to the respective diffusion times *τ*_*Di*_ and the radial dimension *r*_*0*_ of *V*_*obs*_ by *D*_*i*_ = *r*_*0*_^*2*^ */* *(4* *τ*_*Di*_ *)*. By inserting *D*_*i*_ into the Stokes‐Einstein equation, hydrodynamic radius can be calculated as *R_h_ = (k_B_∙T)/(6∙π∙η∙D)*, here, *k*_*B*_ is the Boltzmann constant, *T* is the temperature, and *η* is the viscosity of the solvent. Brightness was calculated as *B = I/N*, *I* is the average fluorescence intensity, and the number of dyes per CSB was calculated by the brightness of CSB divided by the brightness of Alexa Fluor 647. Alexa Fluor 647 was used for the calibration of the confocal volume *V* *_obs_*.

(1) $$G(\tau )=1+\left[1+\frac{f_{T}}{1-f_{T}}e^{-\tau \tau_{T}}\right]\frac{1}{N}\sum_{i=1}^{m}\frac{f_{i}}{\left(1+\frac{\tau }{\tau_{D,i}}\right)\sqrt{1+\frac{\tau }{S^{2}\cdot \tau_{D,i}}}}$$

Synthesis of Sarcosine *N*‐Carboxyanhydride (Sar‐NCA). The synthesis of sarcosine NCA was adapted from the literature and modified.^[^ ^42^ ^]^ Initially, vacuum‐dried sarcosine (21.5 g, 240 mmol, 1.0 eq.) was placed into a pre‐dried, three‐neck, round‐bottom flask. Subsequently, 300 mL of absolute tetrahydrofuran (THF) was added under a continuous flow of nitrogen, and 23.3 mL (190 mmol, 0.8 eq.) of diphosgene was slowly introduced via a syringe. The colorless suspension was gently refluxed for 2 h, resulting in a clear solution. The solution was then subjected to a continuous flow of dry nitrogen for an additional 2 h, with the outlet connected to two gas washing bottles filled with aqueous NaOH solution to neutralize phosgene. The solvent was removed under reduced pressure, yielding a brownish oil as a crude reaction product. The oil was further dried under reduced pressure to obtain an amorphous solid, free of phosgene and HCl, as confirmed by testing against a silver nitrate solution. The crude product was redissolved in 40 mL of THF and precipitated with 300 mL of dry hexane. After filtration under a dry nitrogen atmosphere and drying in a stream of dry nitrogen for 60–90 min, the product was subjected to a high vacuum for 2 h in a sublimation apparatus. Sublimation of the crude product was performed at 80 °C and 10^−3^ mbar. The purified product (137 mmol, 58% yield, colorless crystallites; melting point: 103 °C) was transferred in a Schlenk tube that was handled in a glovebox, and stored at −80 °C.

^1^H NMR (400 MHz, CDCl_3_): *δ* [ppm] = 4.14 (2H, s, ─CH_2_─CO─), 3.04 (3H, s, ─CH_3_).

Synthesis of γ‐(benzyl)‐l‐glutamate *N*‐carboxyanhydride (Glu(OBn)NCA). The synthesis of Glu(OBn)NCA was adapted from the literature and modified.^[^ ^50^ ^]^ Initially, vacuum‐dried benzyl‐ester protected glutamine (15.3 g, 64.5 mmol, 1.0 eq.) was placed into a pre‐dried, three‐neck, round‐bottom flask. Subsequently, 250 mL of absolute tetrahydrofuran (THF) was added under a continuous flow of nitrogen, and 6.3 mL (52 mmol, 0.8 eq.) of diphosgene was slowly introduced via a syringe. The colorless suspension was gently refluxed for 1.5 h, resulting in a clear solution. The solution was then subjected to a continuous flow of dry nitrogen for an additional 2 h, with the outlet connected to two gas washing bottles filled with aqueous NaOH solution to neutralize phosgene. The solvent was removed under reduced pressure, yielding a yellow oil as a crude reaction product. The oil was further dried under reduced pressure to obtain an amorphous solid, free of phosgene and HCl, as confirmed by testing against a silver nitrate solution. The crude product was redissolved in 50 mL of THF and precipitated with 500 mL of dry hexane twice, washed with hexane, dried in a dry nitrogen flow, and finally in a high vacuum. The purified product (11.7 g, 44.4 mmol, 68% yield, colorless needles; melting point: 93–94 °C) was transferred in a Schlenk tube and stored at −80 °C.

^1^H NMR (400 MHz, DMSO‐*d*_6_) *δ* [ppm] = 9.04 (1H, s, ─NH‐), 7.42–7.20 (5H, m, C_6_H_5_), 5.09 (2H, s, ─**CH_2_** ─C_6_H_5_), 4.46 (1H, dd (^3^ *J*_H,H_ = 7.9), 5.5 Hz, ─CO─**CH**─NH─), 2.52‐2.48 (2H, t (^3^ *J*_H,H_ = 7.9), BnO─CO─**CH_2_** ─), 2.15‐1.85 (2H, m, ─**CH_2_** ─CH─).

Synthesis of Azido‐Butyric Acid Pentafluorophenyl Ester. The γ‐azido butyric acid (2 g, 15.5 mmol, 1.0 eq.) was dissolved in pre‐dried THF. After adding triethylamine (2.15 mL, 31.0 mmol, 2.0 eq.), the solution was stirred for 30 min at room temperature. Pentafluorophenol trifluoroacetate (5.2 mL, 30.0 mmol, 2.0 eq.) was added dropwise with a syringe, and the reaction mixture was stirred overnight at room temperature. Completion of the reaction was verified using thin‐layer chromatography (TLC). After THF was distilled, the remaining solid was extracted with water three times. The organic phase was dried with MgSO_4_, and dichloromethane was evaporated off the product. The product was purified by column chromatography.

^1^H NMR (400 MHz, CDCl_3_): *δ* [ppm] = 3.46 (2H, *t*, *J* = 6.5 Hz, ─CH_2_─CH_2_─**CH_2_** ─N_3_), 2.80 (2H, t, *J* = 7.2 Hz, ─CH_2_─**CH_2_** ─CH_2_─N_3_), 2.05 (2H, m, ─**CH_2_** ─CH_2_─CH_2_─N_3_).

^19^F NMR (376. MHz, CDCl_3_): *δ* [ppm] = ‐152.71 (2F, d, o‐CF), ‐157.66 (1F, t, p‐CF), ‐162.11 (2F, t, m‐CF).

Core–shell brush syntheses. Both brush polymers were prepared from poly‐l‐lysine macroinitiators (*DP* ≈250), following the procedure described below for pLys_250_ *‐g*‐[pGlu(OBn)_5_‐*b*‐pSar_50_(N_3_)] using the γ‐(benzyl)‐l‐glutamic acid NCA and sarcosine NCAs.

### Synthesis of Poly‐l‐Lysine_250_‐*Graft*‐poly‐γ‐Benzyl‐l‐Glutamate_5_‐*Block*‐Polysarcosine_50_(N_3_) (pLys_250_ *‐g*‐[pGlu(OBn)_5_‐*b*‐pSar_50_(N_3_)], CSB‐S)

The poly‐l‐lysine trifluoroacetate (*DP*_n_ ≈250, *M*_n_ = 61000 g·mol^−1^) macroinitiator (22.5 mg, 0.37 µmol, 1.0 eq.) was weighed into a predried Schlenk tube and dried by azeotropic distillation with toluene in vacuo overnight. Next, the macroinitiator was dissolved in freshly degassed dry DMF (1.0 mL) and cooled to 0 °C. Then 1.2‐fold excess DIPEA based on amine groups was added (19.3 µL, 0.11 mmol) to neutralize TFA salts. After stirring for 30 mins, γ‐(benzyl)‐l‐glutamic acid NCA (121.7 mg, 0.46 mmol, 1250 eq.) was added as a stock solution in dry DMF. The polymerization was performed at an overall mass concentration of *β* = 100 g·L^−1^ and monitored by IR spectroscopy and HFIP‐SEC. After 5 days, full conversion was observed and sarcosine NCA (550.0mg, 4.6 mmol, 12500 eq.) was added as a stock solution at 100 g·L^−1^ in dry DMF. After 5 days, full conversion of Sar‐NCA was observed by IR spectroscopy, and 1.2‐fold excess azido‐butyric acid pentafluorophenyl ester on amine groups was added (32.6 mg, 0.11 mmol) in DMF and stirred for 2 days at room temperature. The final azide‐functionalized core–shell brush polymer was dispersed in MQ water and centrifuged with Amicon Ultra Centrifugal Filters (100 kDa, 4000 g, 10 × 10 min). After lyophilization, a colorless product was obtained (Yield: 363 mg, 85%).

^1^H NMR (400 MHz, DMSO‐*d*_6_): δ [ppm] = 7.5–7.0 (23 H, m, ─CH_2_─**C_6_H_5_** ─), 5.2–4.8 (10 H, m, ─**CH_2_** ─C_6_H_5_─), 4.5‐3.6 (106 H, br, ─CO─**CH**─NH─ and ─NCH_3_─**CH_2_** ─CO), 3.0–2.5 (150 H, br, ─CH_2_─CH_2_─**CH_2_‐**N_3_, ─**CH_2_** ─NH─CO─, ─N**CH_3_** ─CH_2_─CO─), 2.5–1.5 (br, ─CH_2_─**CH_2_** ─CH_2_─N_3_, CH─**CH_2_** ─**CH_2_** ─CO), 1.5‐0.5 (br, ─**CH_2_** ─CH_2_─CH_2_─N_3_, ─CH─**CH_2_** ─**CH_2_** ─**CH_2_** ─CH_2_─NH─).

^1^H NMR (400 MHz, D_2_O): δ [ppm] = 7.5–7.0 (br, ─CH_2_─**C_6_H_5_** ─), 4.6–4.0 (95 H, br, ─**CH_2_** ─C_6_H_5_─, ─CO─**CH**─NH─ and ─NCH_3_─**CH_2_** ─CO), 3.4–3.2 (2 H, m, ─CH_2_─CH_2_─**CH_2_** ─N_3_), 3.2–2.6 (150 H, br, ─**CH_2_** ─NH─CO─, ─N**CH_3_** ─CH_2_─CO─), 2.6–1.7 (br, ─CH_2_─**CH_2_** ─CH_2_─N_3_, CH─**CH_2_** ─**CH_2_** ─CO), 1.7–1.2 (br, ─**CH_2_** ─CH_2_─CH_2_─N_3_, ─CH─**CH_2_** ─**CH_2_** ─**CH_2_** ─CH_2_─NH─).

### Poly‐l‐Lysine_250_‐*Graft*‐Poly‐γ‐Benzyl‐l‐Glutamate_25_‐*Block*‐Polysarcosine_204_(N_3_) (pLys_250_ *‐g*‐[pGlu(OBn)_25_‐*b*‐pSar_204_(N_3_)], CSB‐L)

^1^H NMR (400 MHz, DMSO‐*d*_6_): δ [ppm] = 7.5–7.0 (126 H, m, ─CH_2_─**C_6_H_5_** ─), 5.2–4.8 (48 H, m, ─**CH_2_** ─C_6_H_5_─), 4.5–3.6 (400 H, br, ─CO─**CH**─NH─ and ─NCH_3_─**CH_2_** ─CO), 3.0–2.5 (562 H, br, ─CH_2_─CH_2_─**CH_2_** ─N_3_, ─**CH_2_** ─NH─CO─, ─N**CH_3_** ─CH_2_─CO─), 2.5–1.5 (br, ─CH_2_─**CH_2_** ─CH_2_─N_3_, CH─**CH_2_** ─**CH_2_** ─CO), 1.5–0.5 (br, ─**CH_2_** ─CH_2_─CH_2_─N_3_, ─CH─**CH_2_** ─**CH_2_** ─**CH_2_** ─CH_2_─NH─).

### Preparation of Dual Centrifuged Brush, Hydrophobic Drug‐Loaded CSB

The dual‐centrifuged brush was prepared from 1 mg mL^−1^ brush solution by dissolving 1 mg solid brush into MQ water. Then, 200 µL 1 mg mL^−1^ brush solution was added to a PCR vial and processed with a dual centrifuge machine (DC) (Hauschild SpeedMixer, Hamm, Germany) for 1 h at 2500 rpm, 4 °C to have a dual‐centrifuged brush.^[^ ^60^ ^]^ The drug‐loaded complexes were prepared from dispersing brush (1 mg mL^−1^) and various concentrations of hydrophobic drugs with chloroform into a PCR vial, which was then left to evaporate overnight in a fume hood. After evaporation, 200 µL MQ water was added to the vial to re‐dissolve the blended brush and drugs. The mixture was then processed under the same DC conditions as a dual‐centrifuged brush to form a drug‐loaded complex.

### Loading Efficiency and Loading Amount

The drug‐loaded complexes were filtered with 0.22 µm PTFE filter (Pall Corporation, New York, United States), and sixfold diluted with MQ water. Samples were placed in a 20 °C incubator and UV absorbance of a sixfold diluted complex was detected by UV–vis (Spectrophotometer 8453, Agilent, Japan). MQ water was set as background. The absorbance curve of the complex was processed by SpectraGryph 1.2 and normalized by GraphPad Prism 10. The maximum absorbance peak value was used to calculate the concentration of the complex, based on the calibration curve determined before. The drug‐loading efficiency and amount were calculated in the following formulas (2) and (3):

(2) $$Loading\;amount=C_{\mathrm{test}}\times \;V\times M_{w}$$

(3) $$Loading\;efficiency\;\left(\%\right)=\frac{w_{test}}{w_{weight}}\times \;100\%$$

C_test_: concentration of complex calculated based on the determined calibration curve.

V: sample volume (200 µL).

*M_w_*: molecular weight of hydrophobic drug.

w_test_: loading amount (obtained from formula 2) of hydrophobic drug.

w_weight_: weighing the weight of hydrophobic drug.

### Drug Release

The release of the drug from the complexes was performed at 37 °C in PBS (no calcium chloride, no magnesium chloride) or 2% BSA solution (2 g bovine serum albumin in 100 mL PBS) (Figure S12, Supporting Information). The concentration of complexes was quantified by the formula as mentioned. The complexes solution (200 µL, 30 µm approximately) was injected into a dialysis membrane (molecular weight cut‐off: 3.5 kDa) (Thermo Fisher Scientific, Landsmeer, The Netherlands) and stirred against 50 mL PBS or 2% BSA solution (50 rpm, 37 °C). 50 mL medium was replaced at defined time points and collected. They were acidified by adding 0.1% trifluoroacetic acid (TFA) (v/v) in advance. The cartridge (Sep‐Pak C18 classic cartridge, Waters, Milford, United States) was activated by 3 mL 90/10 methanol/water with 0.1% TFA (v/v/v) buffer, balanced by MQ water with 0.1% TFA (equilibrium and desalination buffer) in MQ water (v/v) before 50 mL acidified sample solution passing by. After steps of acidification, activation, and balance, samples were condensed and desalinated by passing through a cartridge. Recovery experiments indicated an estimated 25% sample loss during this cartridge processing step (Figure S9, Supporting Information). A 2 mL equilibrium buffer was utilized to complete the loading sample solution. The released drug was initially flushed with 1 mL of acetonitrile: H₂O (1: 1), and the remaining solution in the cartridge was also collected, resulting in a total volume of 1.2 mL of released drug solution sample. Each sample was analyzed by UPLC machine (Waters Acquity UPLC equipped with an Acquity UV detector and a Waters BEH C18 1.7 µm (2.1 × 50 mm) column, with 80% buffer B (acetonitrile with 0.1% TFA) and 20% buffer A (MQ water with 0.1% TFA) as a condition. The concentration of the drug in each sample was determined using a standard calibration curve. To calculate the total amount of released drug, the measured concentration was extrapolated to account for the 25% sample loss that occurred during the cartridge processing step (Figure S9, Supporting Information).

### Visualization of Cellular Uptake and Cell Viability Assay—Cell Culture

U‐87 MG cells were cultured in a medium with components of DMEM/F12 and 10% fetal bovine serum (FBS), 2 mm l‐glutamine, penicillin/streptomycin (P/S) (100 IU mL^−1^ penicillin and 100 µg mL^−1^ streptomycin). The cells were passaged with 0.25% trypsin/EDTA with a confluency of 80–90%. The cell was cultured in an atmosphere of 37 °C with 5% CO_2_.

Visualization of cellular uptake: U‐87 MG cells were seeded in a 96‐well cell plate (Greiner Bio‐One GmbH) at a density of 10 000 cells per well and incubated for 24 h at 37 °C, 5% CO_2_. Afterward, cells were rinsed with PBS twice and cultured with different groups. In each well, 25 µL CSB‐S/DAS complex solution was diluted with 175 µL fresh culture medium to final concentrations of 5 µm DAS and 0.125 mg mL^−1^ CSB‐S. CSB‐S stock solution and DAS stock solution were diluted with PBS and fresh medium to match the brush or DAS concentrations in the drug‐brush complex respectively. After 4 h incubation, cells were rinsed and fixed with 4% paraformaldehyde (PFA) for 20 min, followed by incubation with Hoechst 33342 (1 mg mL^−1^ in fresh culture medium) for 10 min at room temperature. Now, cells were washed with PBS twice and incubated in a fresh culture medium at 4 °C until confocal imaging. Images would be captured with a Leica confocal microscope with 10× lens. Image analysis and processing were performed by Image J software.

### Half Maximal Inhibitory Concentration (*IC_50_)* Test

U‐87 MG cells were cultured at 37 °C with 5% CO_2_ and seeded at a density of 5 000 cells per well in F‐bottom 96‐wells plates (Greiner Bio‐One, Alphen aan den Rijn, The Netherlands) 24 h in advance. The cells were incubated with free drug (in DMSO) and brush (in MQ water), and drug‐loaded complexes, respectively. The final concentration of the free drug ranged from 100 to 0.0001 µm. The brush concentrations used in dual centrifugation were in the range of 10^−2^–10^−4^ mg mL^−1^, which matched the brush concentration in the highest concentration drug‐loaded complex. 20 µL drug‐loaded complexes were added to each well to a final concentration of 1–0.0001 µm. After 72 h of incubation, the culture medium was replaced with 200 µL of MTT solution (1 mg mL^−1^). The plates were covered with aluminum and incubated for 3 h. With the addition of 200 µL DMSO, MTT crystals were dissolved by placing plates on a shaking bed. The samples were measured at l_max_ = 590 nm and l_0_ = 690 nm (as background signal), respectively on Spark Microplate Reader (Tecan Austria GmbH). The absorbance of the samples was calculated by excluding the influence from the background. The cell viability was then calculated with the following methods and graphed in GraphPad Prism 10.

### Statistical Analysis

Experiments were performed at least three times on independent days. Significance between the means of the two groups was tested using 2‐way ANOVA (except for exclusively mentioned) with the software GraphPad Prism 10. Asterisks indicate statistical significance: ^*^ *p* < 0.05; ^**^ *p *< 0.01; ^***^ *p* < 0.001, ^****^ *p *< 0.0001.

## Conflict of Interest

The authors declare no conflict of interest.

## Supporting information



Supporting Information

## Data Availability

The data that support the findings of this study are available from the corresponding author upon reasonable request.
