# Supplementary material for: Polypept(o)ide‐Based Core–Shell Bottlebrush Polymers: A Versatile Platform for Drug Encapsulation
Source: Macromol Biosci. 2025 Apr 22;25(7):2500083. doi: 10.1002/mabi.202500083 (PMC12259402; doi:10.1002/mabi.202500083)
Supplement: Supplementary file 1 — Supporting Information [file MABI-25-2500083-s001.docx]

**Table of Contents**

**Page**

[**Figure S1.** ^1^H NMR (A) and DOSY NMR (B) of pLys_250_*-g*-[pGlu(OBn)_5_-*b*-pSar_50_(N_3_)] (CSB-S) in DMSO-*d*_6_ and D_2_O 3](#_Toc189402500)

[**Figure S2.** ^1^H NMR (A) and DOSY NMR (B) of pLys_250_*-g*-[pGlu(OBn)_25_-*b*-pSar_204_(N_3_)] (CSB-L) in DMSO-*d*_6_ and D_2_O 5](#_Toc189402501)

[**Figure S3.** Preparative SEC elugram for CSB-L purification by collecting fractions 6](#_Toc189402502)

[**Figure S4.** HFIP-SEC monitored enzymatic degradation of CSB-L with protease 7](#_Toc189402503)

[**Figure S5.** Fluorescence correlation spectroscopy analysis 8](#_Toc189402504)

[**Figure S6.** AFM images of CSB-S 9](#_Toc189402505)

[**Figure S7.** Quantification of CSB/DAS complex formulation 10](#_Toc189402506)

[**Figure S8.** Calibration curve of DAS detected by UPLC 11](#_Toc189402507)

[**Figure S9.** Recovery rate of DAS through SPE cartridge 12](#_Toc189402508)

[**Figure S10.** Confocal images of U-87 MG cells after 4 h incubation. 13](#_Toc189402509)

[**Figure S11.** Cell viability assay of CSB-S 14](#_Toc189402510)

[**Figure S12.** Flow chart of release experiment. 15](#_Toc189402511)

**Supplementary Figures**


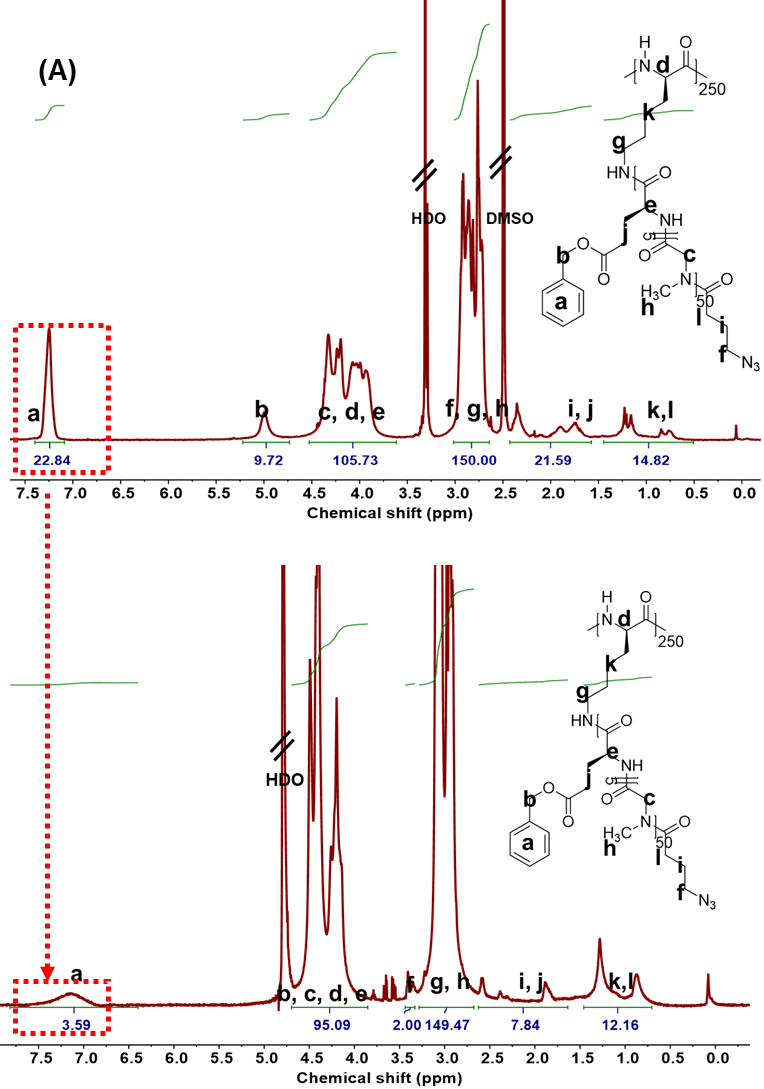


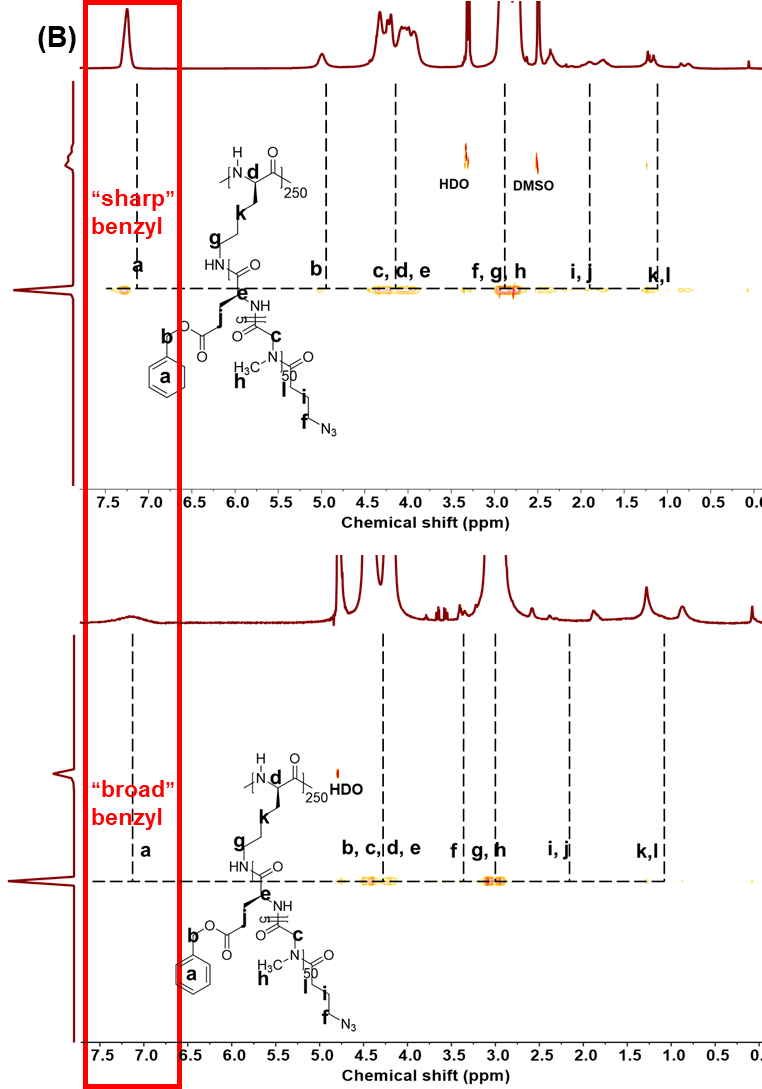


**Figure S1.** ^1^H NMR (A) and DOSY NMR (B) of pLys_250_*-g*-[pGlu(OBn)_5_-*b*-pSar_50_(N_3_)] (CSB-S) in DMSO-*d*_6_ and D_2_O, with the protons assigned and integrated (400 MHz).


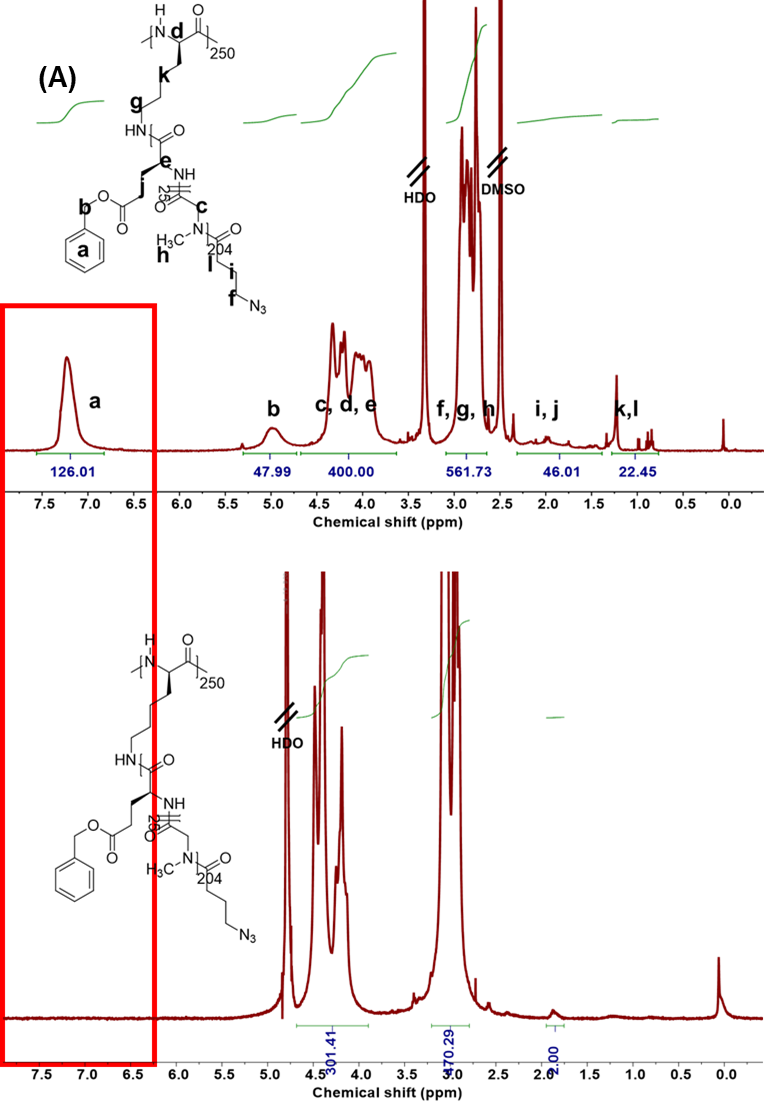


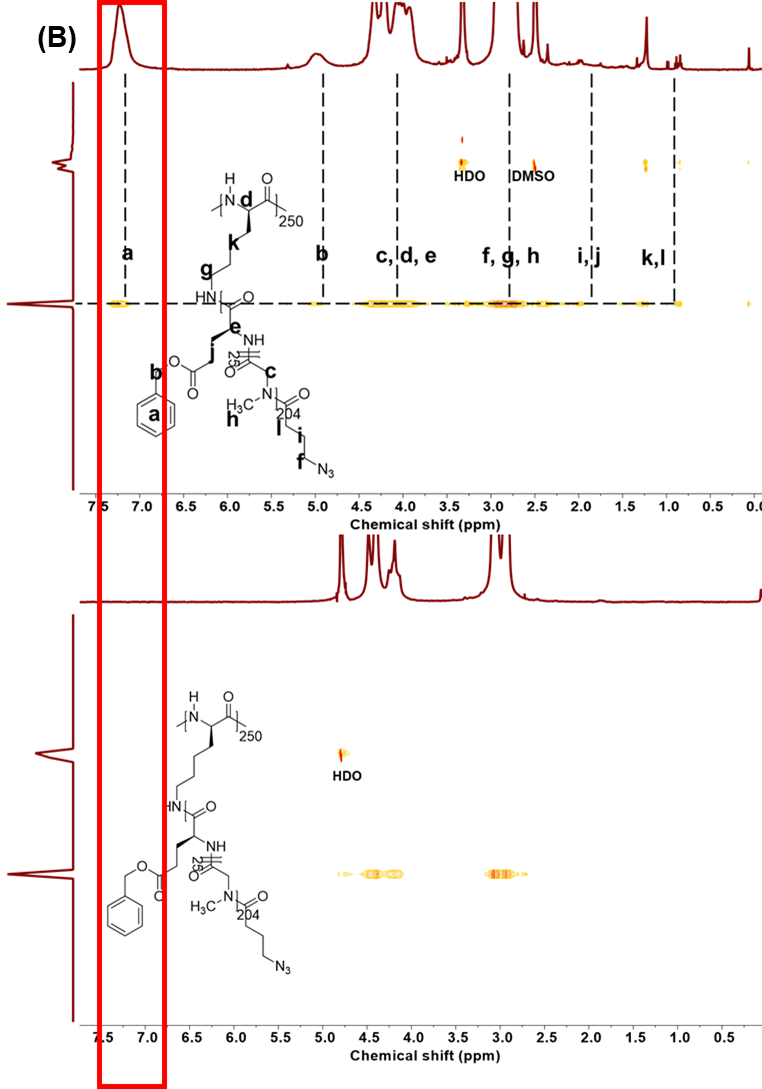


**Figure S2.** ^1^H NMR (A) and DOSY NMR (B) of pLys_250_*-g*-[pGlu(OBn)_25_-*b*-pSar_204_(N_3_)] (CSB-L) in DMSO-*d*_6_ and D_2_O, with the protons assigned and integrated (400 MHz).

**
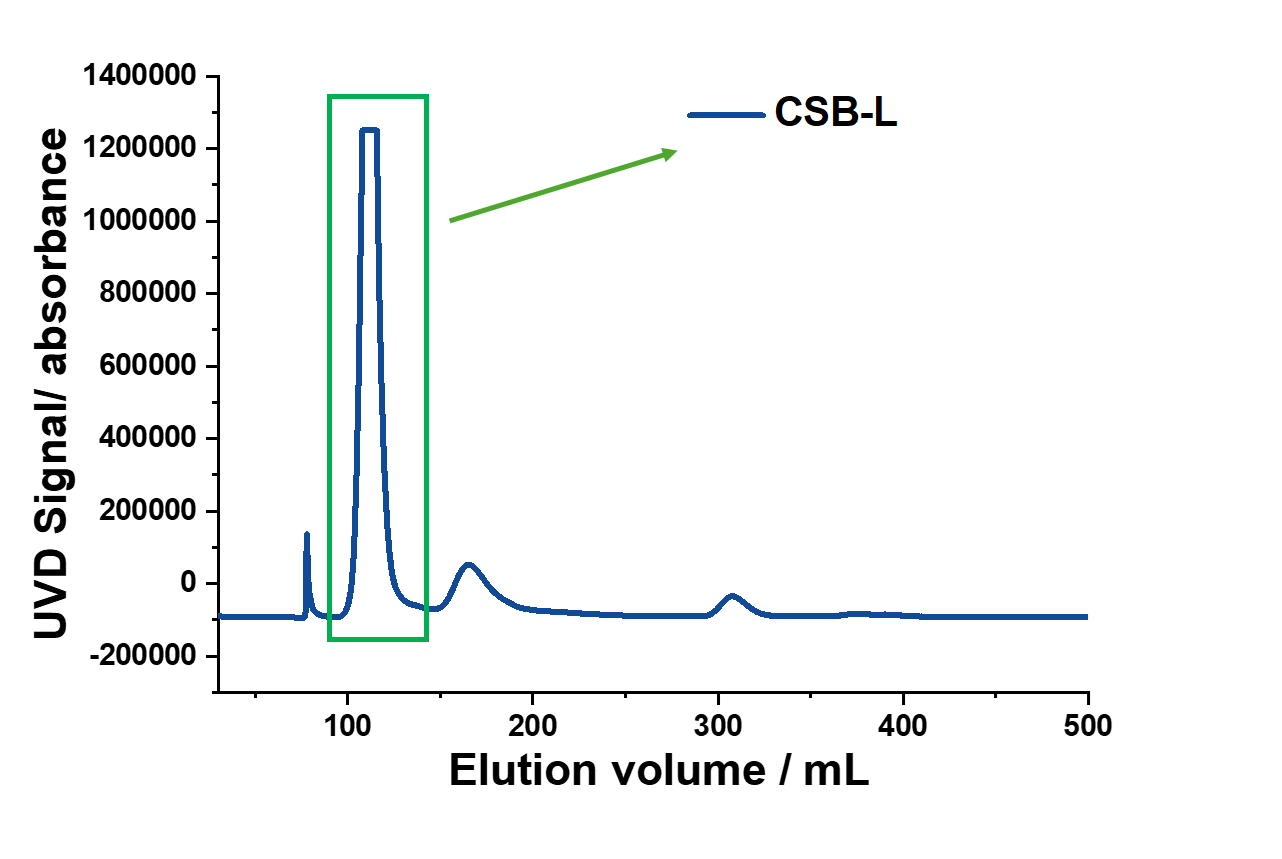
**

**Figure S3.** Preparative SEC elugram for CSB-L purification by collecting fractions.

**Figure S4.** HFIP-SEC monitored enzymatic degradation of CSB-L with protease coincubation in PBS at 37 °C at different time points.


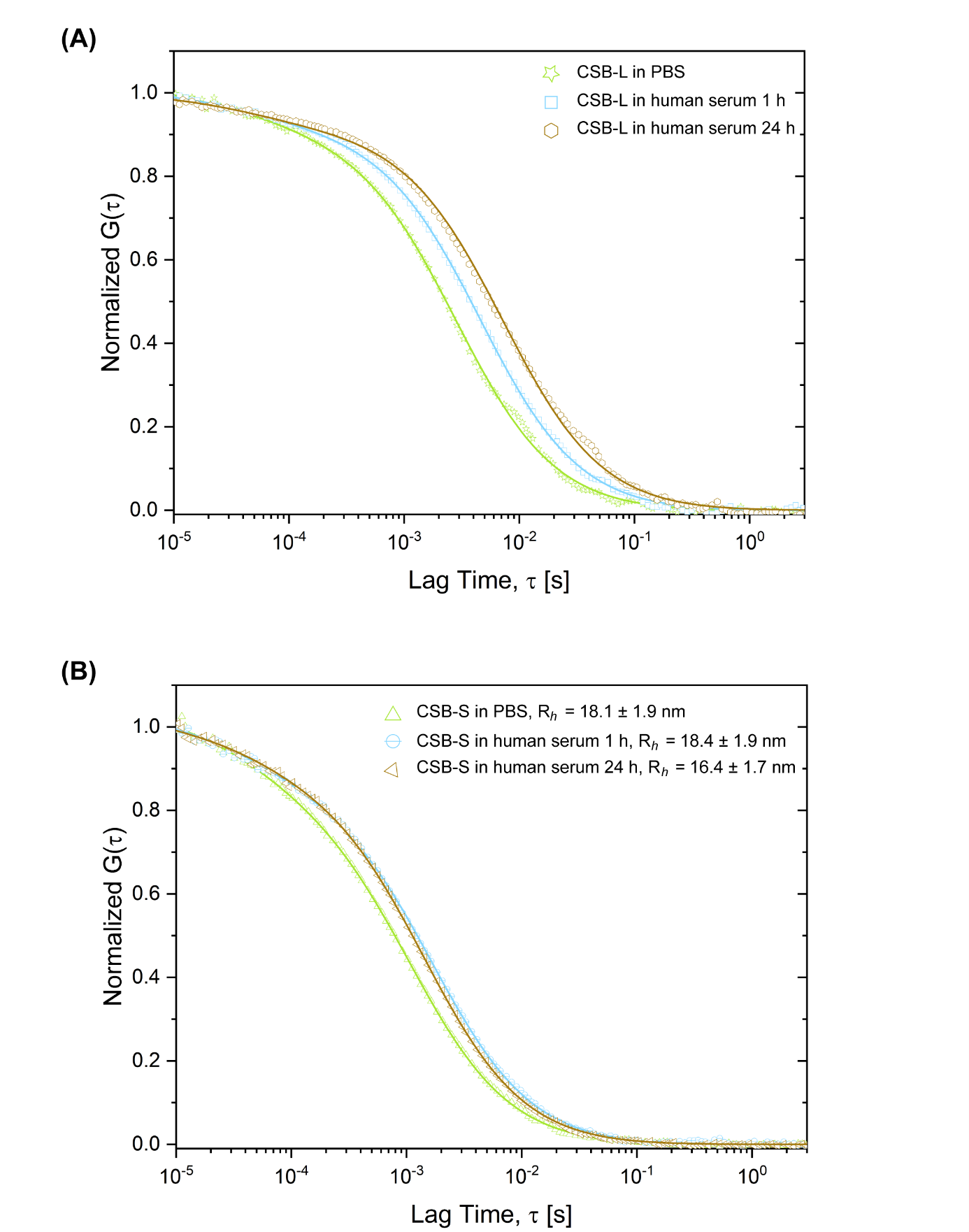


**Figure S5.** Autocorrelation analysis of Alexa Fluor 647-labeled CSB-L (A) and CSB-S (B) in PBS or undiluted human serum after incubation for 1 h and 24 h, measured by fluorescence correlation spectroscopy.


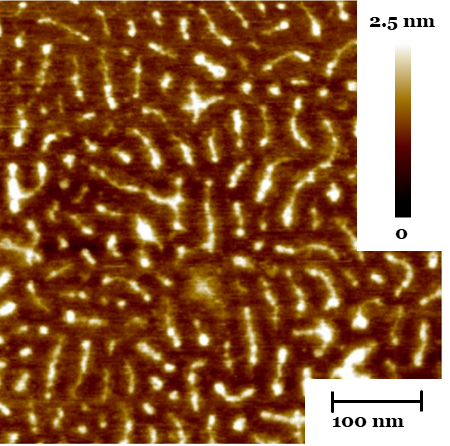


**Figure S6.** Representative AFM images of CSB-S on a mica surface with a lateral scale bar of 100 nm and z-range of 2.5 nm.


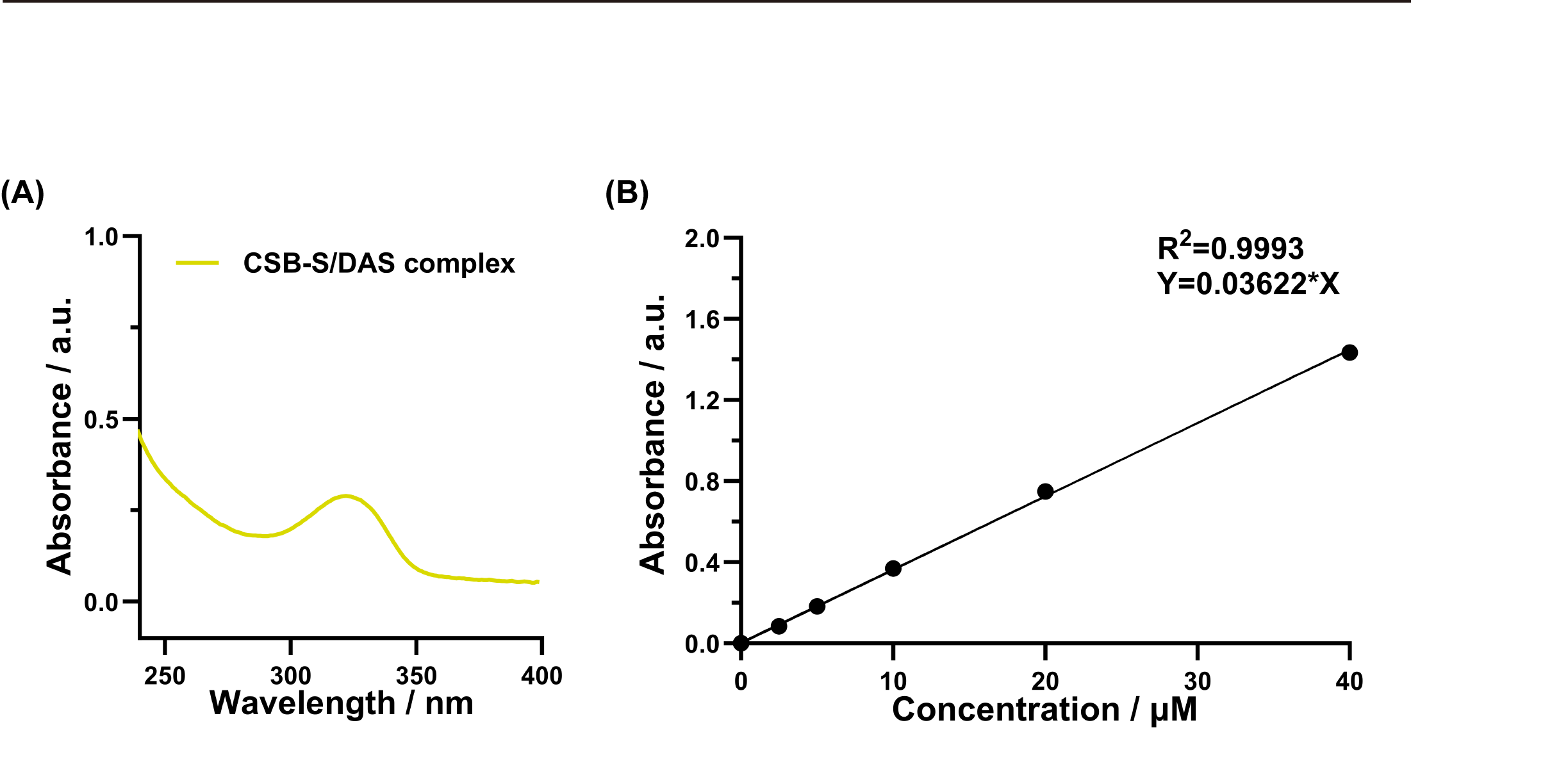


**Figure S7.** UV-vis analysis of CSB-S/DAS complexes at 324 nm (A). Calibration curve of DAS in DMSO by plotting UV absorbance (324 nm) against concentration of DAS (B).

**
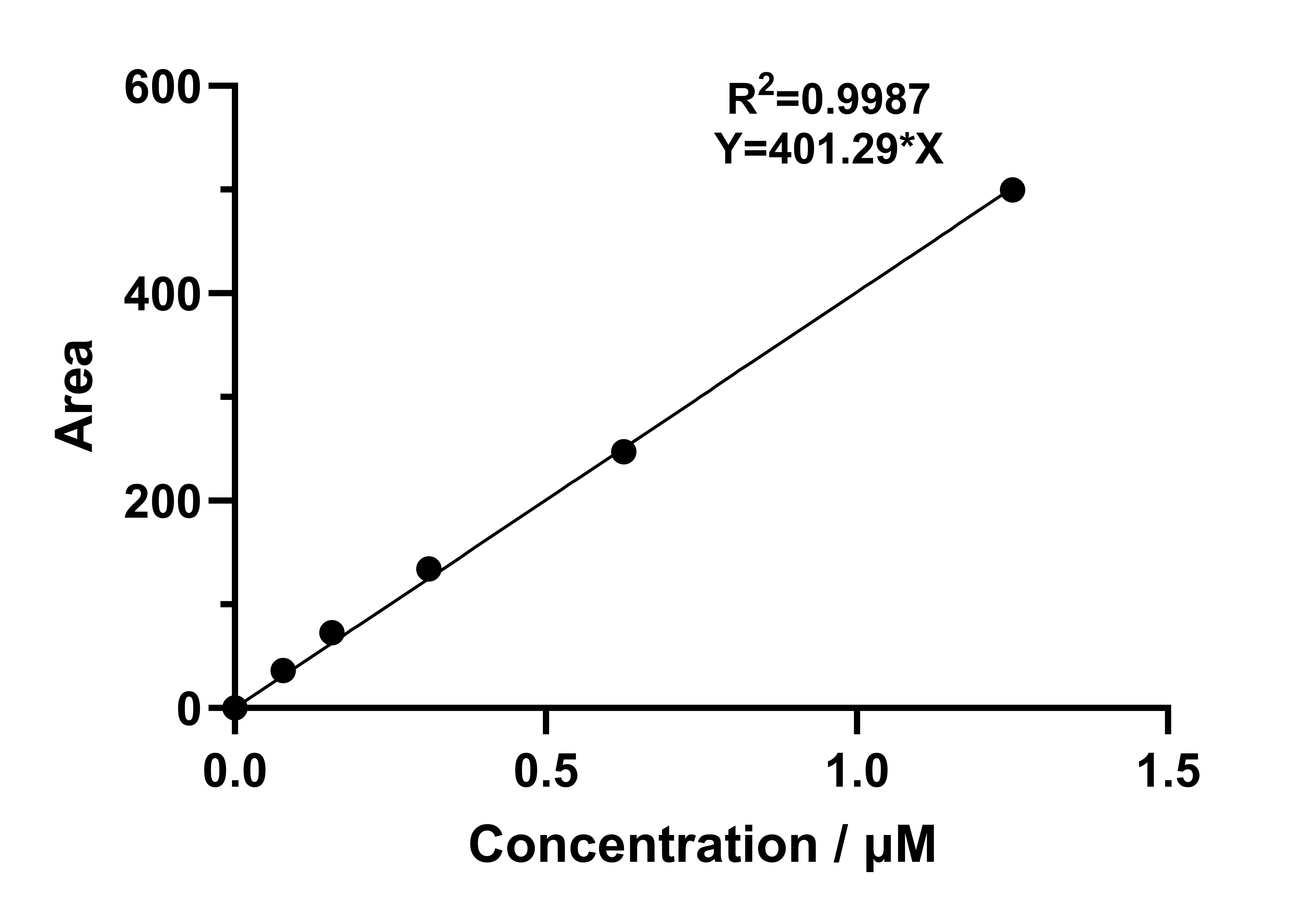
**

**Figure S8.** Calibration curve of DAS dissolved in methanol: MQ water (1:1), determined by UPLC at 1.25 μM, 0.625 μM, 0.312 μM, 0.156 μM, 0.078 μM, with UV detector (324 nm).

**
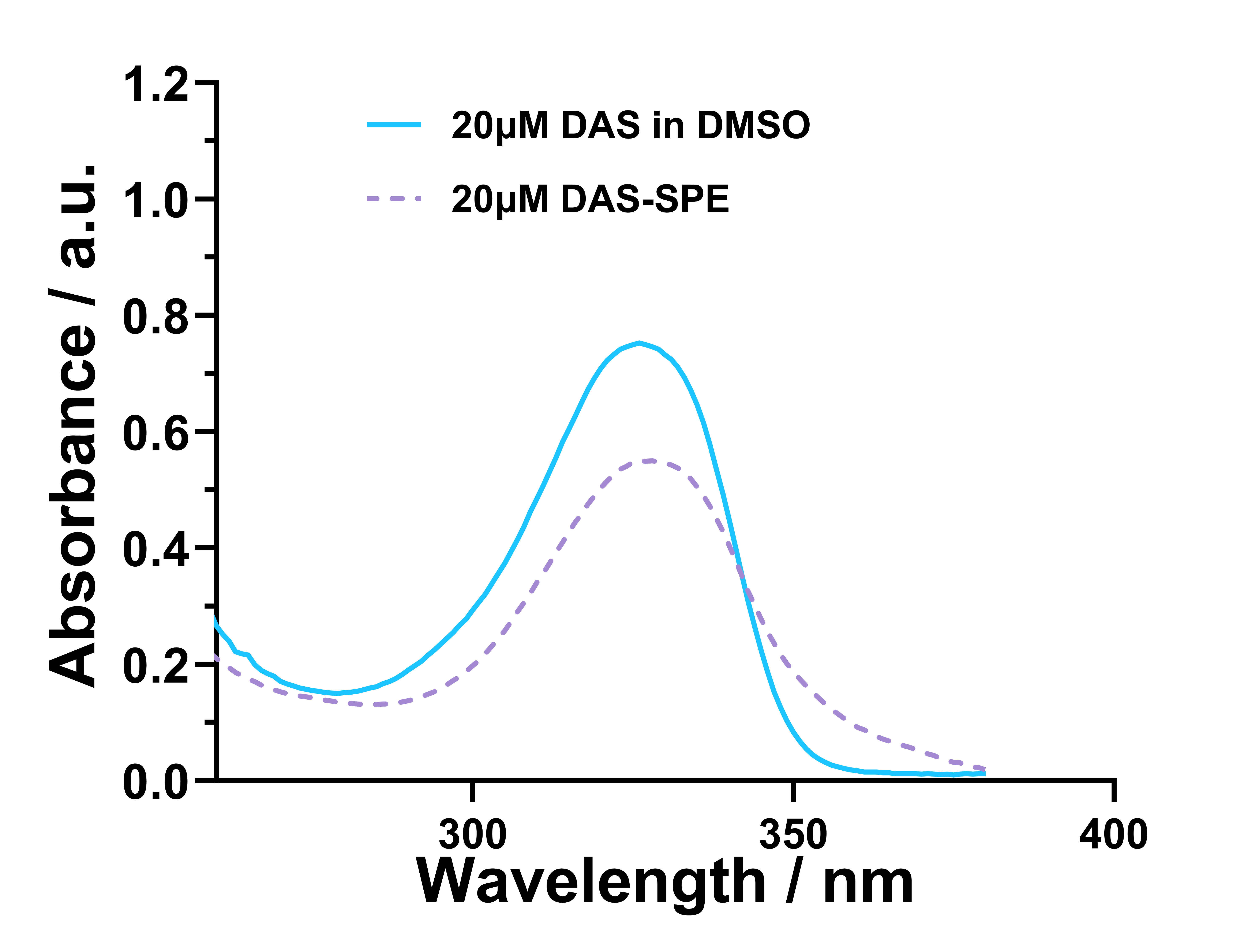
**

**Figure S9.** UV absorbance of DAS following solid phase extraction (SPE) and in DMSO (at equal concentration 20 µM). Based on the UV absorbance (324 nm), approximately 75% DAS was obtained after SPE.


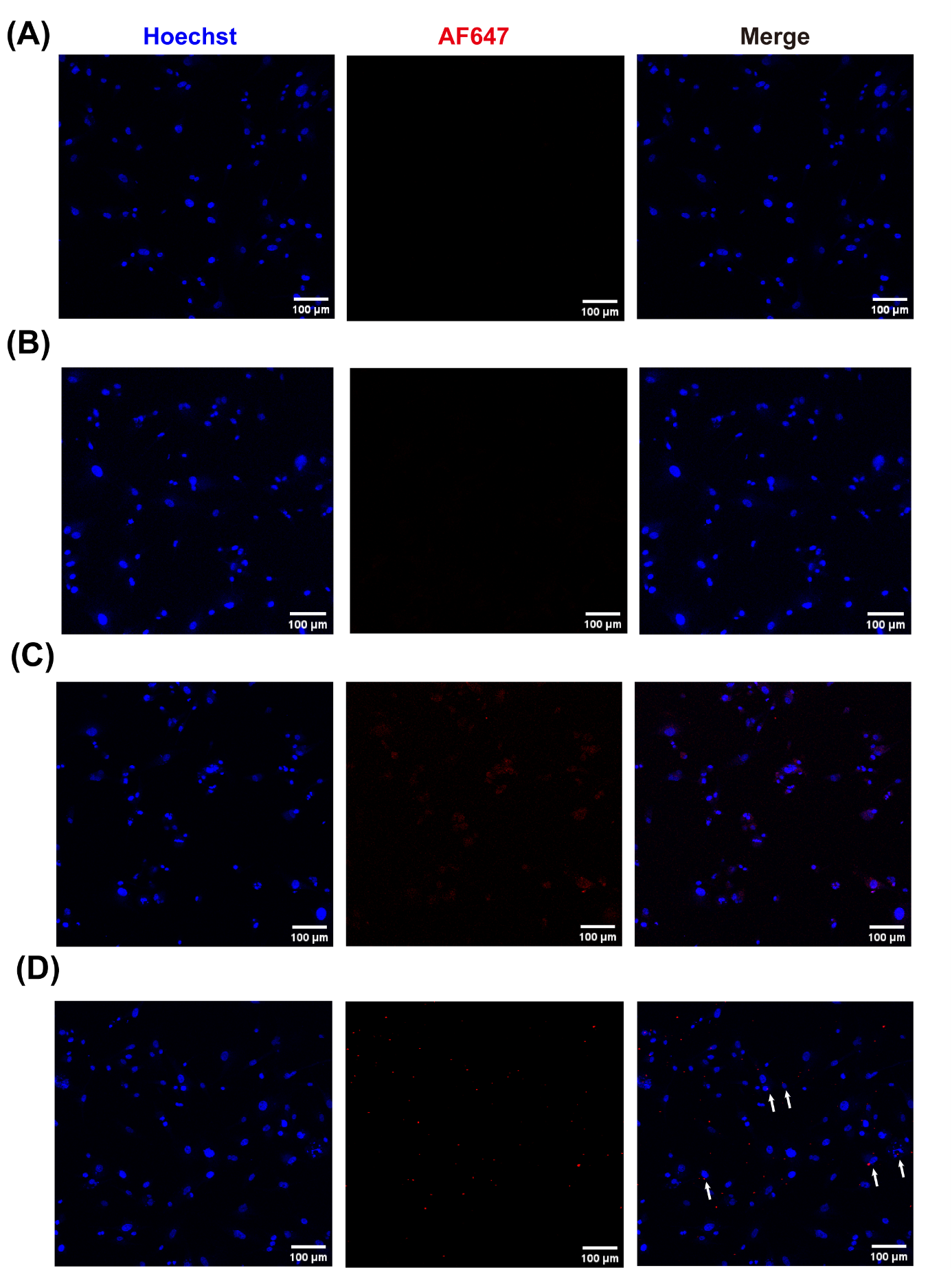


**Figure S10.** Representative confocal images of U-87 MG cells after 4 hours’ exposure to the following conditions: PBS (A), 5 μM DAS (B), and DAS/CSB-S complex (final concentration: 5 μM DAS, 0.125 mg/mL CSB-S) (C), and 0.125 mg/mL AF647-labeled CSB-S (D). Scale bar: 100 µm.


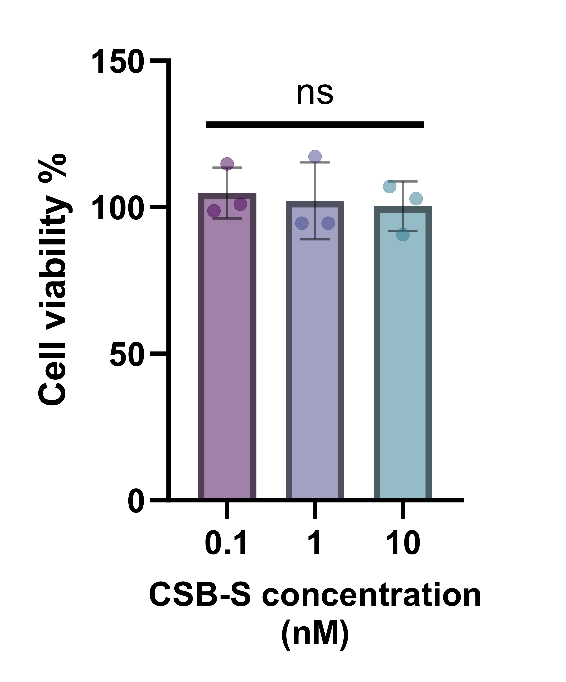


**Figure S11.** Cell viability was assessed using MTT assay after 72-hour exposure to CSB-S. All experiments were implemented in triplicates.


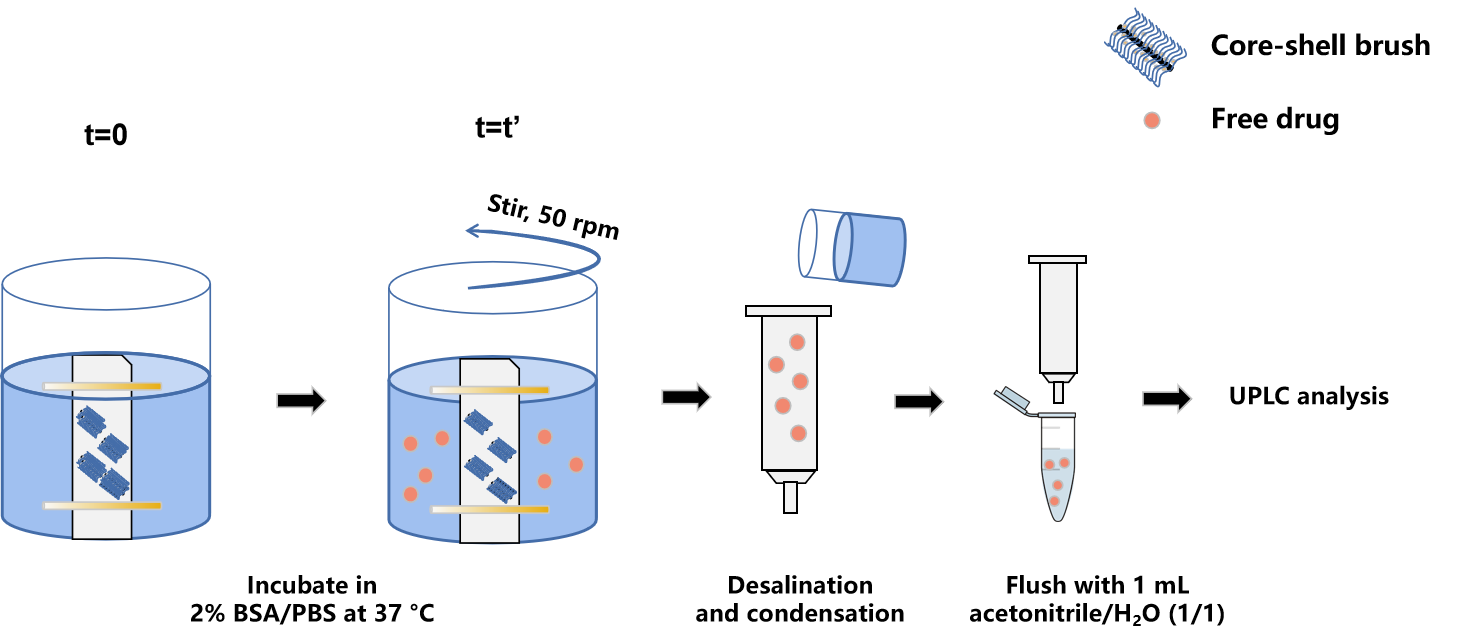


**Figure S12.** Flow chart of release experiment.
